# PFClust: a novel parameter free clustering algorithm

**DOI:** 10.1186/1471-2105-14-213

**Published:** 2013-07-03

**Authors:** Lazaros Mavridis, Neetika Nath, John BO Mitchell

**Affiliations:** 1 Biomedical Sciences Research Complex and EaStCHEM School of Chemistry, Purdie Building, University of St Andrews, North Haugh, St Andrews, KY16 9ST, Scotland, UK

## Abstract

**Background:**

We present the algorithm PFClust (Parameter Free Clustering), which is able automatically to cluster data and identify a suitable number of clusters to group them into without requiring any parameters to be specified by the user. The algorithm partitions a dataset into a number of clusters that share some common attributes, such as their minimum expectation value and variance of intra-cluster similarity. A set of *n* objects can be clustered into any number of clusters from one to *n*, and there are many different hierarchical and partitional, agglomerative and divisive, clustering methodologies available that can be used to do this. Nonetheless, automatically determining the number of clusters present in a dataset constitutes a significant challenge for clustering algorithms. Identifying a putative optimum number of clusters to group the objects into involves computing and evaluating a range of clusterings with different numbers of clusters. However, there is no agreed or unique definition of optimum in this context. Thus, we test PFClust on datasets for which an external gold standard of ‘correct’ cluster definitions exists, noting that this division into clusters may be suboptimal according to other reasonable criteria. PFClust is heuristic in the sense that it cannot be described in terms of optimising any single simply-expressed metric over the space of possible clusterings.

**Results:**

We validate PFClust firstly with reference to a number of synthetic datasets consisting of 2D vectors, showing that its clustering performance is at least equal to that of six other leading methodologies – even though five of the other methods are told in advance how many clusters to use. We also demonstrate the ability of PFClust to classify the three dimensional structures of protein domains, using a set of folds taken from the structural bioinformatics database CATH.

**Conclusions:**

We show that PFClust is able to cluster the test datasets a little better, on average, than any of the other algorithms, and furthermore is able to do this without the need to specify any external parameters. Results on the synthetic datasets demonstrate that PFClust generates meaningful clusters, while our algorithm also shows excellent agreement with the correct assignments for a dataset extracted from the CATH part-manually curated classification of protein domain structures.

## Background

In pattern recognition, data analysis is used for predicting the behaviour of an unseen test dataset. Amongst problems of this kind, two different types can be clearly distinguished. The first is supervised learning or classification, where a labelled training dataset with known categories is involved. The second kind is unsupervised learning or clustering, where no prior information is available for grouping the dataset. The objective of a clustering algorithm is to partition the given data into mutually exclusive and meaningful [1] clusters; this can provide a better understanding of the natural structure of the data. Semi-supervised [2] classification, which combines strategies from both supervised and unsupervised methods, has also grabbed attention in various fields of research as it requires less human effort and gives better accuracy [3] than unsupervised learning. In this paper, we focus our attention on the challenges faced by clustering algorithms [4,5].

There are numerous clustering algorithms discussed in the literature, traditionally clearly distinguished as either hierarchical [6] or partitional [7]. Hierarchical methods group the objects together layer by layer, based on the closeness of the data points as measured by suitable similarity or distance metrics. Hierarchical clustering sequentially partitions the dataset, either by successively dividing an initial single cluster in divisive methods, or by joining together initially unlinked objects in agglomerative algorithms. In a hierarchical method, once two objects are clustered together they remain together at all subsequent levels of the scheme with fewer clusters. Contrastingly, partitional clustering algorithms, such as k-means, do not have a layer by layer structure and objects may sometimes move from one cluster to another.

The k-means method iteratively assigns each point to the cluster whose centroid is closest to it, recalculates the cluster centroids, and reassigns the points. This process continues until the assignments no longer change at each iteration. k-means tends to generate approximately equally sized clusters, minimising intra-cluster distances; however, its preference for globular clusters and its failure to reproduce clusters of complex shape are limitations [5].

### Determining the number of clusters

Automatically determining the number of clusters is a major problem in clustering. A set of *n* objects can be clustered into any number *k* of clusters 1 ≤ *k* ≤ *n* by any of the methods we have discussed. Identifying the optimal number of clusters involves computing a range of different numbers of clusters *k*, with the objective of finding the best value of *k* that gives the optimum clustering. However, there is no agreed or unique definition of optimum in this context. Using internal and external validation measures as described in Handl *et al*. [5], one could design a protocol for reaching a decision on the best *k*. A gap statistic [8] addressed this issue by acting as an internal validation measure, and has been applied in bioinformatics [9]. Though in principle it is not hard to design a workflow to find the best *k*, in practice this is not commonly done. This is partly because there is no consensus as to which of the many different possible measures should be used to compare clusterings with different numbers of clusters, a more difficult problem than the comparison of clusterings with the same *k*. This adds to the difficulty of choosing the best clustering method for finding the structure of a novel dataset.

### Validation

Validation [4,8] plays an important role in deciding the number of clusters, as well as in assessing the performance of the clustering algorithm. Cluster validation is designed to evaluate and compare clustering algorithms by their ability to analyse a dataset. There are many different validation measures. Internal measures like Silhouette width [9] and Dunn Index [10,11] depend on the inherent structure of the data [12]. External measures, such as the Rand Index [13] commonly used to evaluate the noise in biological data, depend on comparison with an externally known gold standard classification of the objects.

Here we propose a novel clustering algorithm, PFClust (**P**arameter **F**ree **Clust**ering), suitable for use where no prior information about the number of clusters is given. As input, only similarity scores within the dataset are required, and evaluation of the clustering is part of the algorithm. A previous study by Akoglu *et al*. [14] designed a parameter free graph clustering algorithm to find cohesive clusters, PICS. They have shown the efficiency of their method using real-world datasets including data from YouTube and Twitter. Our method shares the property of being parameter-free, but is aimed at classifying objects rather than graphs.

As the availability of biological information accelerates, it is necessary to find the natural structure or patterns in data in order to understand biological questions. In bioinformatics, grouping proteins based on sequence [1] or structure is a very common task. Classification of novel proteins [15] can be performed by using pattern recognition approaches, built on the assumption that some underlying characteristics are considered, while clustering proteins into superfamilies and families. There are numerous classification schemes for protein sequences including PIR-PSD [16], a freely available database of protein sequence classification mostly applied for functional annotation, and Pfam [17], a classification of functional protein domains based on hidden Markov models and multiple sequence alignments.

Extending these ideas to three-dimensional (3D) protein structure provides the interesting task of clustering and classifying protein domain folds. During the early 1990s the Protein Data Bank (PDB) [18] held only a few thousand 3D crystal structures, and several initiatives for protein fold classification were proposed with CATH [19] and SCOP [20] being the best known. These were based on either manual curation (SCOP) or computer-aided manual curation (CATH). Common to both approaches is that the human curator has the final word in the classification decision. With the exponential growth of the number of 3D high resolution structures deposited in the PDB during the last decade [21], reaching 87,085 structures at the beginning of 2013, the rate-limiting manual part of the curation process restricts our capacity to understand the full structural diversity of proteins. Hence it would be ideal if a fully automated process could classify protein domains and cluster them into structurally similar groups.

## Methods

Here we describe a partitional algorithm that uses the idea that each cluster can be represented as a non-predetermined distribution of the intra-cluster similarities of its members. The algorithm partitions a dataset into a number of clusters that share some common attributes, such as their minimum expectation value and variance of intra-cluster similarity. It is an agglomerative algorithm, meaning that it starts with separated objects and progressively joins them together to form clusters. PFClust is heuristic in the sense that it cannot be described in terms of optimising any single simply-expressed metric over the space of possible clusterings. Nonetheless, we demonstrate that, over a number of validations on test datasets, it produces clusterings that closely reflect the structure of the test data, and outperforms many well established algorithms in this regard. We have taken a number of design decisions to optimize the algorithm with respect to time efficiency and result stability.

Since we represent each cluster as the distribution of the similarities between its members, we need to have a clustering criterion in order to separate different clusters and find the cluster structure. The criterion used in this algorithm is the expectation value of the similarity distribution between cluster members. In order to select a suitable threshold, we will need a good approximation to the distribution of mean intra-cluster similarities for all possible clusterings. We can then select the most appropriate threshold value given an internal validation measure. The number of unique clusterings for a dataset with *n* points when we do not know the number of clusters, *O*(*n*), *a priori* is the Bell number:

$$o\left(n\right)=\sum_{k=0}^{n}\frac{1}{k!}\sum_{j=0}^{k}\left({\left(-1\right)}^{k-j}\left({}_{j}^{k}\right)j^{n}\right)$$

where *k* is the number of clusters, inclusive of singletons. This number grows rapidly with the number of points in the dataset. Hence, instead of attempting an exhaustive search of all possible clusterings, we perform a random sampling, where we randomly decide the number of clusters and randomly assign the initial distribution of points amongst clusters. This sampling approach is necessary for efficiency, but introduces a random element to the algorithm. In order to optimize some of our design decisions for the algorithm, we have internally validated using a synthetic dataset of 1500 two-dimensional (2D) vector points.

### Algorithm

**Figure 1:**
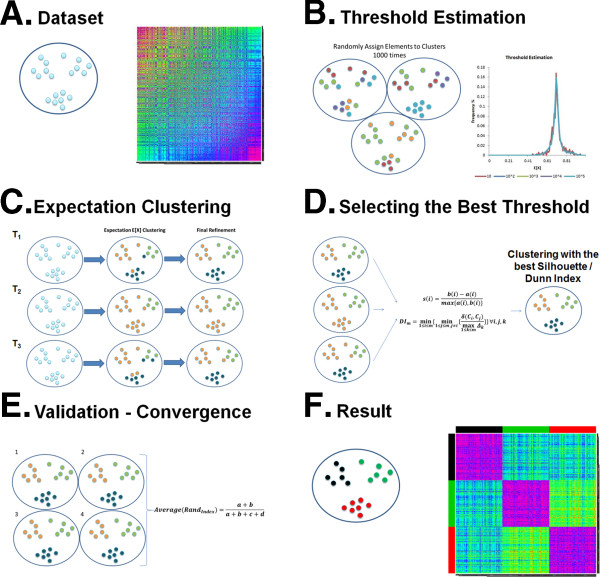
**Visual representation of the algorithm.** This figure provides a visual representation of the algorithm as a number of different steps **(A** to **F)**.

The clustering algorithm consists of two parts. The first part is the randomization, and the second part incorporates both the threshold selection and the actual clustering. Thus, in the first part (panel B of Figure 1), 20 thresholds are estimated (*T*_*1*_,…, *T*_*20*_) by a randomization process. In the second part (panels C & D of Figure 1), each threshold is used to cluster the data, and the best threshold is selected. This whole process, incorporating both randomization and threshold selection, is carried out four times (panel E of Figure 1). If the four resulting clusterings do not agree, the algorithm replaces the least successful of the four runs with a fresh attempt and repeats until convergence. Figure 1 shows a graphical representation of the clustering algorithm.

### Threshold estimation

**Figure 2:**
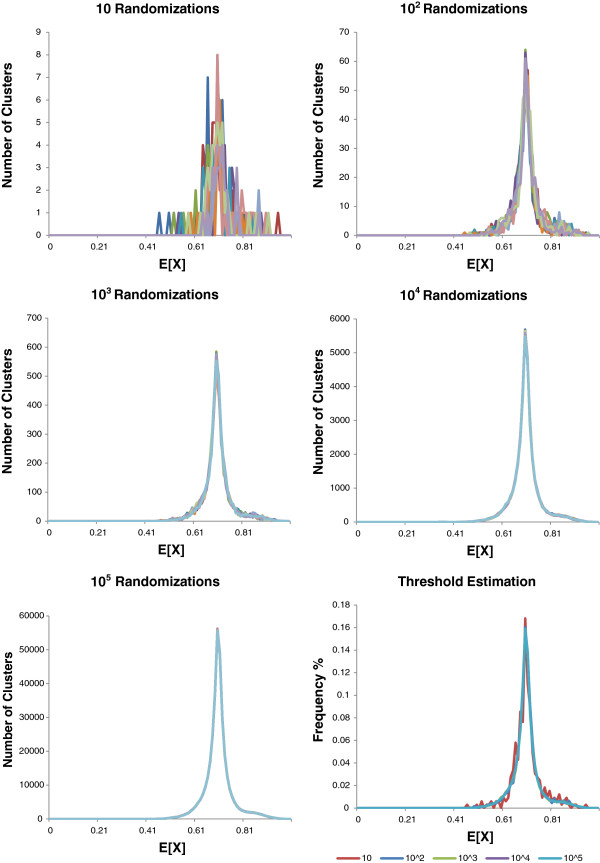
**Distribution of the E[X] according to the number of randomizations *N*.** For each value of *N* (*N* = 10, *N* = 10^2^, *N* = 10^3^, *N* = 10^4^ &*N* = 10^5^), the randomization process was run independently ten times and the distribution of the E[X] was plotted.

Given a dataset Ω = {α_*1*_,…,α_*n*_} with *n* members, the threshold values are estimated by multiple random clusterings of the data. Firstly, a random number of clusters *k* (where *1*≤ *k* ≤ *n*) is chosen and each data point α is initially randomly placed in one of the clusters. Now let *X*_*i*_ be the distribution of the similarities between the elements of cluster *i*. The mean intra-cluster similarity, which is the expectation value of the distribution *X*_*i*_, is defined so as to exclude self-similarities and can be calculated as:

$$E\left[X_{i}\right]=\frac{1}{\left(\begin{array}{c} n_{i}\\ 2 \end{array}\right)}\overset{n_{i}}{\underset{j=2}{\sum }}\sum_{q=1}^{j-1}s_{\alpha_{j},\alpha_{q}}$$

where *X*_*i*_ is the *i*^*th*^ cluster, *n*_*i*_ is the number of members of that cluster and $s_{\alpha_{j},\alpha_{q}}$ is the similarity between elements α_j_ and α_q_. In order to estimate an efficient value for *N*, the number of randomizations used for threshold estimation, we selected a dataset and simulated the randomization process. We repeated this ten times for a number of different values of *N*. Figure 2 shows that using a small number of randomizations greatly affects the shape and sharpness of the distribution. However, increasing *N* greatly affects the execution time of the algorithm, since the randomization is the most expensive part of the calculation. Hence, as a compromise between accuracy and expense, we selected *N* = 1000 as the number of randomizations, since this is the smallest number which gives an acceptably sharp distribution of mean intra-cluster similarities. The intra-cluster mean similarities, *E*[*X*_*i*_], from every individual cluster across the 1000 randomizations are collated into a single distribution. From this distribution, we retrieve 20 threshold values from the 95% - 100% significance levels, that is the 5% of the clusters with the highest mean intra-cluster similarities. We select the ten values corresponding to the following percentiles {95.00%, 97.50%, 99.00%, 99.14%, 99.29%, 99.43%, 99.57%, 99.71%, 99.86% and 100.00%} and ten further thresholds corresponding to the second to eleventh highest values in the distribution of intra-cluster similarities. Using this number of thresholds provides a way of reducing the random element of our sampling.

### Similarity-based clustering

Now, for each threshold value *T*, the dataset *Ω* is clustered with a similarity-based clustering. We begin with all elements separated and no clusters defined. The two most similar elements in *Ω* are placed together to form the first cluster. For each of the (*n*-*2*) remaining elements, we compute its average similarity *E*[*X*] to the elements already in cluster 1, and we now identify the element with the highest similarity. If this value is larger than *T*, the algorithm considers adding this most similar element to the given cluster. The element is added if and only if the element’s average similarity compared to the members of that cluster is at least *P*% of *T* and the overall *E*[*X*] of the cluster is also larger than *T*. This process is repeated until no new element can be added to the cluster without *E*[*X*] of the cluster falling below *T*. At this stage, a new cluster is formed from the two most similar remaining elements, provided that their similarity exceeds *T*. This process is continued iteratively until all elements of *Ω* have been clustered, or until the remaining elements cannot form a cluster that has an expectation value of intra-cluster similarity greater than *T*.

The *P*% of *T* cut-off was selected as a way to restrict the intra-cluster variation of the similarities since, in a very tight cluster, outlier members could be included because, even if they are distant from the other cluster members, the total *E*[*X*] could still be above *T*. In order to estimate the value of *P*, we performed a number of experiments on our dataset with different *P* values. Table 1 shows the results, from which we see that a value of *P* = 85% of *T* gives the optimal results with respect to the Silhouette width as well as the number of clusters, with multi-member clusters and singletons being shown separately.

**Table 1:** Comparison of different values of P

**P Value**	**Clusters**	**Singletons**	**Silhouette width**	**Avg Std**
T	30	6	0.1719	1.1740
0.95 × T	10	4	0.5240	4.2715
0.90 × T	10	1	0.5650	4.3176
0.85 × T	10	0	0.5961	4.6604
0.80 × T	10	0	0.5955	4.8175
0.75 × T	10	0	0.5955	4.8175

The table summarizes the performance of the different P values for the threshold inclusion rule. The numbers of multi-member clusters and singletons are given separately, so that the total numbers of clusters at each P value are 36, 14, 11, 10, 10, and 10, respectively. The Silhouette width and the average of the standard deviations of the distributions of intra-cluster similarities in each cluster are also shown.

After the data are assigned to clusters, a final refinement step is applied to all points that have an average similarity score less than *T* when compared to the members of the cluster they have been assigned to. Each such point has its average similarity calculated with every cluster and is assigned to the cluster to which it is most similar (if this is not its original cluster, then the process moves the point to a different cluster; this assignment to a cluster is made even if the point’s average similarity to the members of its new cluster is less than *T*).

### Selecting the best threshold

For each of the 20 different *T* values, a clustering has been obtained from the algorithm. For each of these clusterings, the mean Silhouette width (averaged over every point in the dataset) and the Dunn Index of the clustering are computed. The Silhouette width is defined for each element *i* as:

$$s\left(i\right)=\frac{b\left(i\right)-a\left(i\right)}{\max\left\{a\left(i\right),b\left(i\right)\right\}}$$

where *a*(*i*) is the average dissimilarity, where dissimilarity is 1-similarity, of element *i* with all the other elements of the same cluster and *b*(i) the average dissimilarity of element *i* to all the members of the closest neighbouring cluster. In order to take singletons into account, a negative score (−1) is given to each singleton point in the proposed clustering. The Dunn Index is defined as:

$$DI_{m}=\min_{1\leq i\leq m}\left\{\min_{1\leq j\leq m,j\neq i}\left\{\frac{\delta \left(C_{i},C_{j}\right)}{\max_{1\leq k\leq m}\Delta_{k}}\right\}\right\}\forall i,j,k$$

where  *δ*(*C*_*i*_, *C*_*j*_) is the inter-cluster distance between the centres of clusters *i* and *j*, and max _1 ≤ *k* ≤ *m*_Δ_*k*_ is the maximum cluster size in the dataset, where cluster size is defined as the mean distance between all members of the cluster and the cluster centroid. The cluster centroid is the element with the maximum similarity to the other members of the cluster. The Silhouette width is the main factor used in deciding which threshold produces the best clustering, and the Dunn Index is used only as a tie-breaker to decide cases where two or more clusterings have the same Silhouette width.

### Convergence

As mentioned above, the algorithm has a random sampling aspect. In order to increase the probability of ultimately finding the best possible clustering, we choose to repeat the process a number of times. In order to decide a number of repetitions that is time efficient and reduces the probability of runs (of the whole multi-repetition procedure) generating different outputs, we performed a single clustering experiment on our dataset. We ran the algorithm (excluding repetitions) 100 times using a dataset of 1500 2D vectors and, considering the event *A* as “seeing the clustering result with the maximum Silhouette width”, we found *p*(*A*) = *76*%. Therefore, if we run the algorithm only once, we have a 24% probability of not finding the best solution. Ideally, we would like to have a very small probability of such an event. Using four repetitions reduces that probability to 0.3%, which is sufficiently small for our needs. Hence, the whole process is repeated four times and the four different clusterings are retrieved and compared. If all four runs give the same clustering, the algorithm is said to have converged and stops. If not, for each of the six different pairs of clusterings chosen from the four clusterings made, a Rand Index between a pair of clusters is calculated as:

$$\mathrm{Rand}\;\mathrm{Index}=\frac{a+b}{a+b+c+d}$$

where *α* is the number of cases where two elements are members of the same cluster in both clusterings, *b* is the number of cases where two elements are members of different clusters in both clusterings, *c* is the number of cases where a pair of elements are in the same cluster for the first clustering and in different for the second and *d* is the number of cases where a pair of elements are members of different clusters for the first clustering and members of the same cluster for the second clustering. Using all six different pairs of clusterings we calculate the average Rand Index.

We use the Rand Index because it is a widely accepted measure of concordance between different clusterings (here, the four clusterings produced by the four runs) and not as a maximization metric compared to some original classification. If the average Rand Index is high (>0.99), this means that most of the runs report near-identical clusterings with no significant differences. Hence, this is enough for the algorithm to converge and report the clustering with the highest Silhouette width. As mentioned above we want to be very confident in the resulting clusters, therefore a very strict average Rand Index of 0.99 (which allows for a limited number of differences in assignment of borderline cases) is applied as a cut-off. In the case of an average Rand Index less than 0.99, we consider that we have found significantly different clusterings. Then, an instance of the clustering with the lowest (or equal lowest) Silhouette width is removed, even if this outcome has been found two or three times, and another randomization is done. This procedure is repeated until convergence.

### Pseudocode

I. Do four times:

Stage 1: Calculate D (the distribution of E[X]’s).

1. Do the specified “randomization” 1000 times:

i. Randomly select a number of clusters *k*.

ii. Randomly assign each data point α to a cluster *c*.

iii. ∀ clusters *c*, calculate *E[X]* for the pairwise point-point similarities within *c* and include this value of *E[X]* in *D*.

2. For each of the ten percentiles {95.00%, 97.50%, 99.00%, 99.14%, 99.29%, 99.43%, 99.57%, 99.71%, 99.86% and 100.00%} of the distribution *D* of intra-cluster similarities, and for ten further thresholds corresponding to the second to eleventh highest values, retrieve a threshold value *T*.

Stage 1A: Clustering

i. While any *α* in the dataset remains unclustered:

a. Join the two most similar currently unclustered elements to form a new cluster, provided criteria in b. are met.

b. Calculate average similarity of each currently unclustered data point to the current cluster and keep adding the most similar available data point as a member as long as both:

– *E*[*X*] of the cluster > *T,* and.

– the average similarity of the new member to the existing members of the current cluster > 0.85×*T.*

Stage 1B: Clustering Refinement

ii. ∀ *α* ∈ any *c*, retrieve its average similarity with all the members of its current cluster. If this average similarity < *T* then:

a. If its average similarity with elements of any other cluster is more than that with the parent cluster, move the point *α* to this other cluster.

iii. Measure the Silhouette width, averaged over all points with singletons each contributing −1, and the Dunn Index for the final clustering for this *T* value.

3. Return the *T* value and resultant clustering with the best Silhouette width as the result of the run; in the event of a tie, use the Dunn Index to decide.

II. Repeat until Convergence:

 Stage 2: Convergence (measure the Rand Index between each of the four runs)

1. If average Rand Index amongst all 6 pairs taken from the 4 clusterings ≥ 0.99, return the clustering with the best Silhouette width as the final result (algorithm converged).

2. If this average Rand Index < 0.99, the algorithm has not converged and the clustering with the lowest Silhouette width is discarded and we repeat *Stage 1* a single time to generate a new clustering.

### Validation

In order to validate PFClust, we used a number of synthetic 2D datasets. The first dataset consisted of 3000 2D vectors distributed over 20 groups; for each of these groups, the probability density function falls off with distance from its centre according to a normal distribution. Hence the groups are approximately circular. Each group corresponds to the external gold standard definition of a cluster. We also used subsets of 300 and 450 2D vectors, respectively composed of two and three out of the 20 groups in the 3000 vector dataset. The second dataset consisted of 5000 2D vectors distributed over 15 groups, which vary in shape. Finally, the third dataset consisted of 928 2D vectors distributed over 20 clusters, which all have different member densities. In each dataset, the centres are chosen such that there is no significant overlap between groups, though a handful of outlier points appear within an apparently ‘wrong’ group. We performed three different experiments based on the first dataset, in order to illustrate that the method was not finely tuned for a specific number of clusters or cluster structure. We define similarity as one minus the normalized (such that the most distant point is one unit from the origin of the coordinate system) Euclidean distance between two points. We would therefore expect PFClust to perform optimally where the clusters are approximately circular.

On all our datasets, we run PFClust as well as six other current state-of-the-art algorithms. These are (i) the hierarchical clustering algorithm Hierarchy [6]; (ii) the hierarchical AGlomerative NESting (Agnes) [22], (iii) the partitional k-means clustering algorithm [7], (iv) Clustering Large Applications (Clara) [23], which is based on repeated k-mediods clustering of samples, (v) Density-Based Algorithm for Discovering Clusters in Large Spatial Databases (DBSCAN) [24] and (vi) Model-Based Clustering (Mix Model) [25]. We used available implementations of each of these methods in the statistical software suite R, [26] the relevant packages are listed in Additional file 1: Table S1. These algorithms cannot all be compared on the ability to find the optimal number of clusters. Only PFClust and DBSCAN amongst the methods considered here can do this, and in fact the latter algorithm requires two parameters to be optimised before it decides the number of clusters. Hence, for the five other methods, we will use the externally defined ‘correct’ number of clusters (this definition including singletons in the count of clusters) as a given parameter and compare how well each algorithm clusters the data compared to the original classification. In order to compare the different clustering approaches, we selected the Rand Index as a measure of agreement between the externally known ‘correct’ clustering and that produced by a clustering algorithm.

We ran k-means and Clara 100 times each on every dataset and have selected as the final result for each algorithm the one with the best Silhouette width. For the *epsilon* parameter of DBSCAN, the maximum permitted distance between a point and its closest intra-cluster neighbour, we examined all values between 0 and 1 with a step size of 0.001. We also iterated the *min*-*points* parameter, the minimum number of members allowed in a valid cluster, using all integer values from 1 to 100. This resulted in 10^5^ clustering outputs, from which the one with the maximum Silhouette width was selected.

As an addendum to the main work, we tested the use of the Silhouette width as a characteristic measure from which to decide the correct number of clusters. We ran the deterministic methods once each. We also ran the stochastic Clara and k-means algorithms 100 times each for every number of clusters, k, between 2 and 50. The run with the best Silhouette width for a given algorithm was selected, thus deciding the number of clusters to report.

### Protein fold clustering using polar Fourier expansions

A shape-density superposition algorithm based on Spherical Polar Fourier (SPF) basis functions has recently been published [27,28], in which protein shapes are represented as 3D density functions expressed as expansions of orthonormal basis functions:

$$\rho \left(r,\theta ,\varphi \right)=\overset{N}{\underset{\mathrm{nlm}}{\sum }}\alpha_{\mathrm{nlm}}R_{\mathrm{nl}}\left(r\right)y_{\mathrm{lm}}\left(\theta ,\varphi \right)$$

where *y*_*lm*_ (*θ*, *φ*) are the real spherical harmonics, *N* is the order of the highest polynomial power of the expansion, *R*_*nl*_ (*r*) are radial functions, and α_*nlm*_ are the expansion coefficients which are calculated as described previously [29]. Mavridis *et al*. proposed in the same paper a novel structure-based indexing for existing classification schemes such as CATH [19] and SCOP [20]. Their proposed consensus algorithm works well for only some of the cases it was tested on, because of the structural diversity of a number of protein domains assigned to the same superfamilies [28]. Hence, methods such as SPF would greatly benefit from an automated clustering algorithm, such as PFClust, which could identify the structure of a dataset without any prior knowledge or parameter tuning.

In order to illustrate that PFClust could be used to provide such a clustering using the SPF descriptors, we performed the following study. We randomly selected 11 CATH superfamilies, which had in total 224 non-redundant representative structures, and used the SPF descriptors to calculate the all-against-all similarity matrix of these protein domains. We then used PFClust to cluster the protein domain structures based on these similarities.

## Results and discussion

### Original dataset

**Figure 3:**
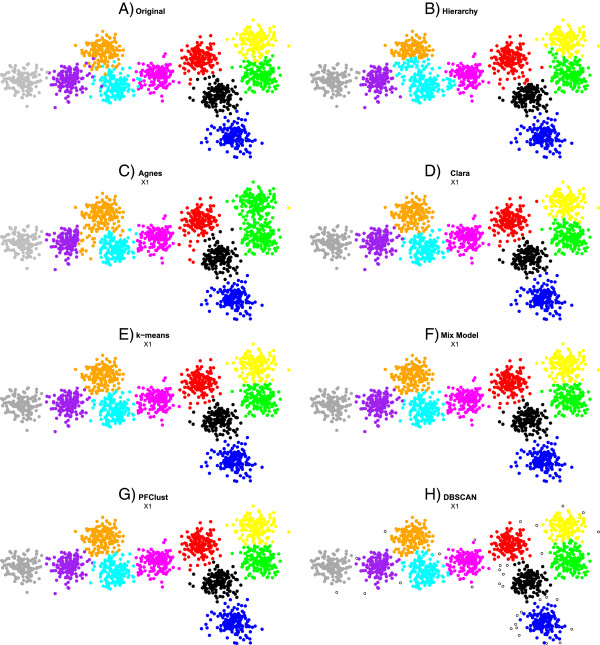
**Comparison of different clustering algorithms.** The 1500 2D vector dataset clustered with all seven different clustering algorithms. In frame **A** the original gold standard clustering is shown. In frames **B**,**C**,**D**,**E**,**F**,**G** and **H,** the proposed clusterings by each method are given.

In this section, we present the resulting clusters from the 1500 2D vector dataset that was used for the general parameter set up of the algorithm. Figure 3 shows the results of each method on the 1500 2D vector dataset; the mismatched points for each method are shown in Additional file 1: Figure S1. From the Rand Index results in Table 2, we see that Agnes has the lowest (worst) score; that is mainly because Agnes joins two groups into a single cluster (green and yellow). Furthermore, we have a singleton as one cluster (yellow point). Rather better levels of performance are achieved by the Hierarchy and DBSCAN algorithms. However, Hierarchy has trouble in correctly assigning a number of the boundary cases for some pairs of clusters (particularly around the boundary between the orange and cyan clusters, and also that between yellow and green) and DBSCAN assigns a large number of points as singletons. Finally, the best performing algorithms are PFClust, Clara, k-means and Mix Model, which correctly identify all clusters and boundaries. Although the Rand Indices for these methods are very good, they fail to reproduce perfectly the original ‘correct’ classification because the original dataset has a number of outlier points that lie closer to the centres of different groups, such as purple elements in the cyan and orange groups. So, for example, a purple element is so coloured because in the original grouping it was generated from the normal distribution used to define the purple cluster. However, it is located significantly closer to the centre of the cyan cluster, and thus we believe that a rational classifier should consider it cyan. Nonetheless, the gold standard we are using means that we count the rational classification as incorrect.

**Table 2:** Comparison of the clustering methods based on popular metrics

**Methods**	**Rand index**							
	**300**	**450**	**1500**	**3000**	**5000**	**CATH**	**Density**	**Average**
**Hierarchy**	0.883	0.942	0.896	0.920	0.981	0.964	0.928	0.931
**Agnes**	0.947	0.973	0.836	0.820	0.976	0.906	0.975	0.919
**Clara**	0.960	0.980	0.952	0.948	0.987	1.000	0.956	0.969
**k-means**	0.960	0.980	0.958	0.966	0.986	0.738	0.901	0.927
**Mix Model**	0.960	0.980	0.959	0.911	0.990	1.000	0.977	0.968
**PFClust**	0.960	1.000	0.958	0.949	0.986	0.996	0.976	0.975
**DBSCAN**	0.973	0.973	0.930	0.921	0.978	0.977	0.688	0.920

The table summarizes the comparison between PFClust and the other six clustering algorithms based on the Rand Index between the clustering predicted by the method in question and the original gold standard clusters.

### Validation

**Figure 4:**
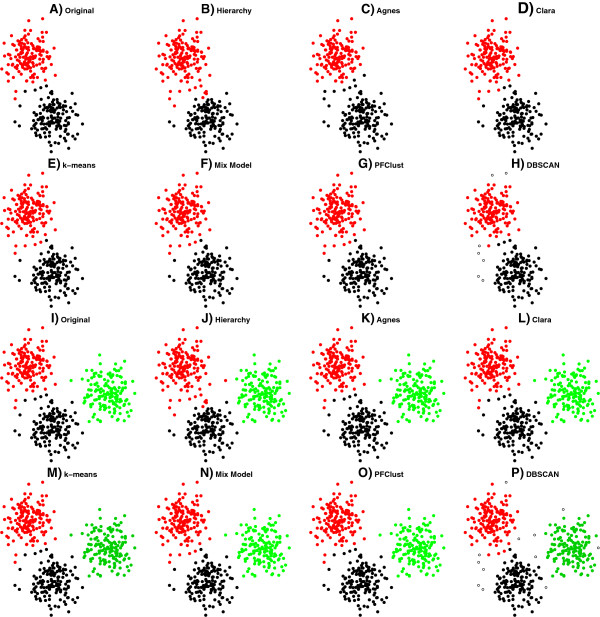
**In frames A (300 2D vectors) and I (450 2D vectors), two of the datasets that were used and their original gold standard clusterings are shown.** In frames **B**, **C**, **D**, **E**, **F**, **G** and **H**, the proposed clusterings by each method for the 300 2D vector dataset are given. In frames **J**, **K**, **L**, **M**, **N**, **O** and **P**, the proposed clusterings by each method are shown for the 450 2D vector dataset.

For the first two experiments with the 300 and 450 2D vectors, all methods perform very well, as shown in Figure 4, with only the Hierarchy clustering performing a little worse in comparison with the rest, as can be seen in Table 2 with the mismatched points for each method being shown in Additional file 1: Figure S2.

Table 2 summarizes the results for all methods on all the different datasets used in this study. We can see from the results that PFClust is the top performing algorithm on average with a mean Rand Index of 0.975, though it perfectly reproduces the original clusters for only one dataset (450 2D vectors).

**Figure 5:**
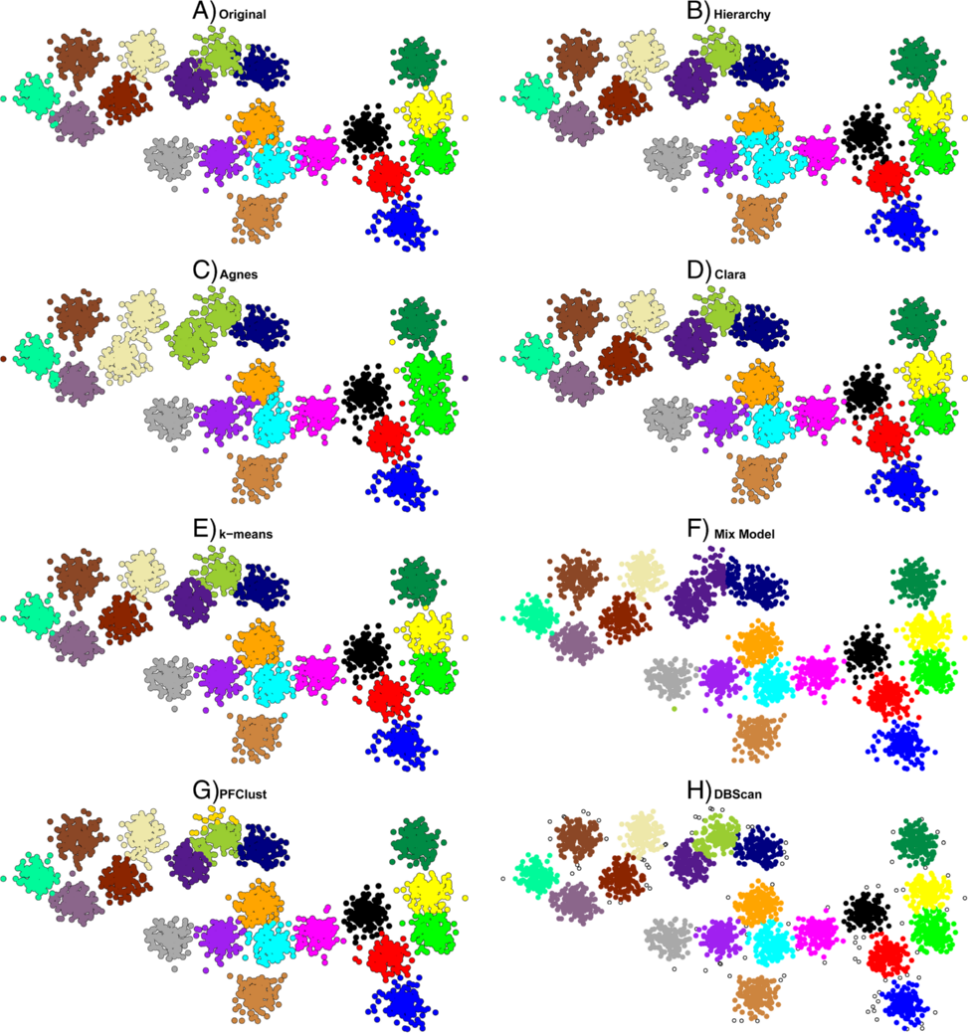
**Comparison of different clustering algorithms.** The 3000 2D vector dataset clustered with all seven different clustering algorithms. For DBSCAN, the open circles denote singletons. In frame **A** the original gold standard clustering is shown. In frames **B**,**C**,**D**,**E**,**F**,**G** and **H**, the proposed clusterings by each method are given.

When we use all 3000 2D vectors, PFClust suggests a different number of clusters from that in the original clustering. In this case, PFClust finds that there are 21 clusters instead of 20 and splits the light green cluster into two, light green and gold. Even though PFClust suggests a different number of clusters, its very good Rand Index means that it still significantly outperforms the other algorithms, except k-means, as can be seen in Table 2. Hierarchy, Clara and DBSCAN achieve good Rand Indices, though with some errors on the borderline cases. Mix Model gives a Rand Index that is only slightly worse, but assigns a singleton as one of its 20 clusters and, as seen in Figure 5, splits the greenish cluster at the top centre of the diagram between its purple and blue neighbours; the mismatched points for each method are shown in Additional file 1: Figure S3. DBSCAN identifies the correct number of multi-member clusters, although as with all datasets it additionally has a large number of points assigned as singletons, shown as open circles in Figure 5.

**Figure 6:**
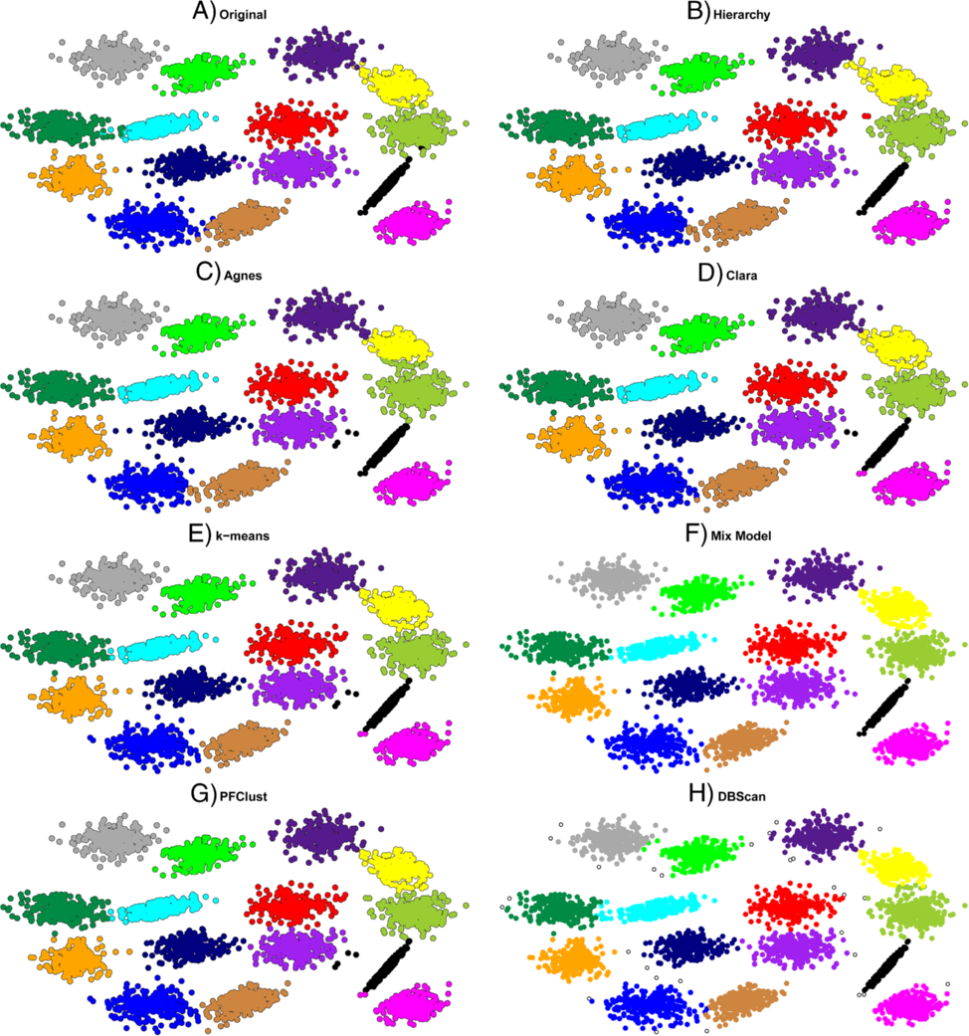
**Comparison of different clustering algorithms.** The 5000 2D vector dataset clustered with all seven different clustering algorithms. For DBSCAN, the open circles denote singletons. In frame **A** the original gold standard clustering is shown. In frames **B**,**C**,**D**,**E**,**F**,**G** and **H**, the proposed clusterings by each method are given.

We also performed another validation study on the bigger dataset of 5000 2D vectors distributed over 15 clusters, in order also to compare the algorithms using a dataset that had different shapes and sizes of clusters, as well as the larger number of data points. As suggested by visual inspection of Figure 6, the Rand Index results in Table 2 show that all algorithms perform very well, with Mix Model having the best Rand Index. The mismatched points for each method are shown in Additional file 1: Figure S4.

Another challenge for algorithms is describing datasets that have different densities of points in their original clusters; the 928 2D vector dataset consists of 20 clusters whose densities vary. This is a subset of the D31 dataset taken from [30], which consists of 31 circular clusters with 100 members each. We then chose a 20 cluster subset of the original dataset and from each cluster we randomly selected a different number of members (varying from 5% to 95%).

**Figure 7:**
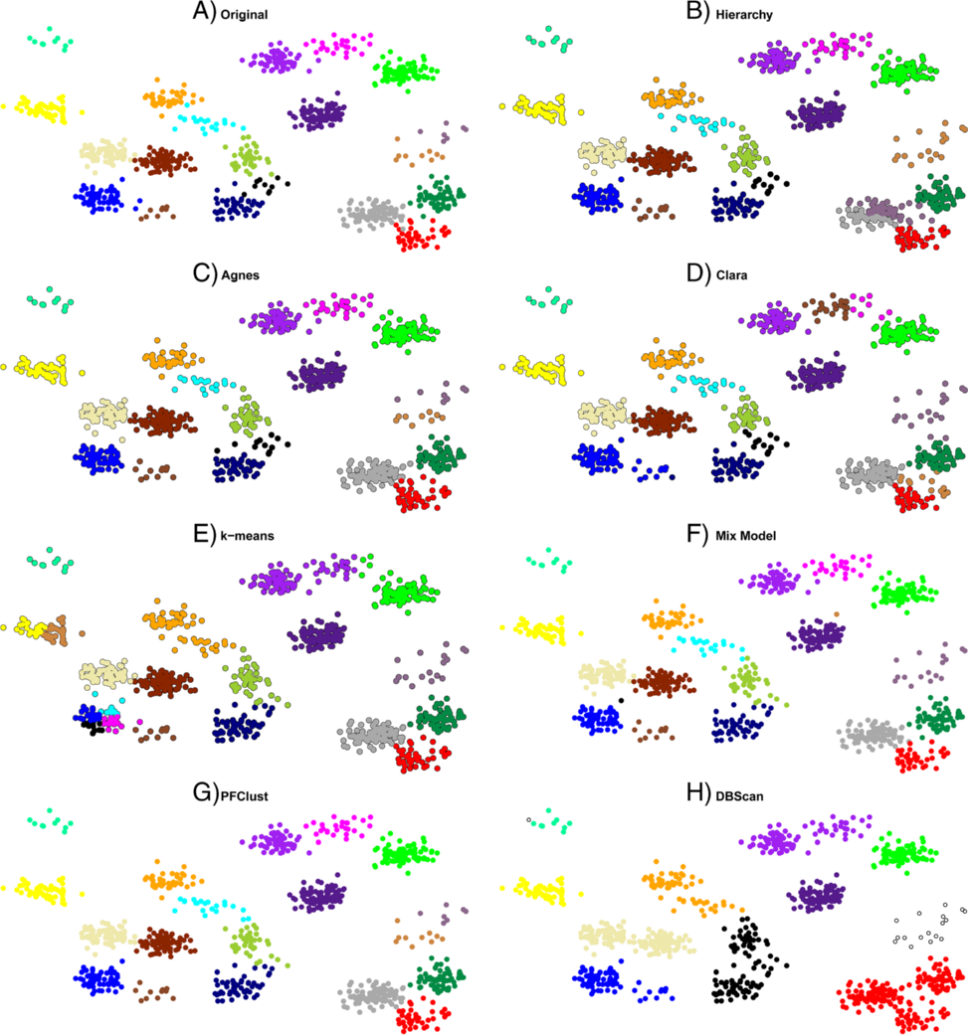
**Comparison of different clustering algorithms.** The 928 2D vector dataset of 20 clusters of varying density, as clustered by the seven different clustering algorithms. For DBSCAN, the open circles denote singletons. In frame **A** the original gold standard clustering is shown. In frames **B**,**C**,**D**,**E**,**F**,**G** and **H**, the proposed clusterings by each method are given.

In this case, we see from Figure 7 that PFClust reports 19 clusters instead of the original 20, integrating the black group into the navy blue and the light green. Of the other methods, only Agnes was able to correctly identify all the groups, but due to the misassignment of some borderline cases it did not achieve a very high Rand Index. DBSCAN has major problems assigning the correct clusters (it finds only 10 multi-member ones), joining many clusters together and again leaving a large number of singletons, as seen in Figure 7; the mismatched points for each method are shown in Additional file 1: Figure S5.

### PFClust – supervised mode

While we have shown PFClust to be a very powerful and accurate algorithm, it does take longer to cluster a dataset than most alternative approaches. Table 3 shows how the running time of the algorithm until convergence varies with the number of data points. It is easy to see that the run time grows rapidly as the dataset becomes larger and that the clustering takes considerable amounts of CPU time in order to converge, with most of it being spent in the randomization process.

**Table 3:** Supervised and Unsupervised timings for PFClust

**Dataset**	**Rand index**	**Randomizations**	**Clustering**	**Total execution time (runs)**
CATH folds	0.996	4.3 s	1.8 s	25 s
300 2D Vectors	0.960	8 s	2 s	40 s
450 2D Vectors	1.000	25 s	8 s	2 m 38 s
1500 2D Vectors	0.958	13 m 22 s	5 m 47s	1 h 22 m
3000 2D Vectors	0.949	2 h 24 m 27 s	1 h 14 m 38 s	12 h
5000 2D Vectors	0.986	11 h 35 m 30 s	5 h 20 m 20 s	81 h 50 m 30 s
Density Dataset	0.976	6 m 20 s	2 m 15 s	36 m
1500 Supervised	0.958	-	1 m 50 s	2 m 57 s
3000 Supervised	0.951	-	12 m 20 s	20 m 39 s
5000 Supervised	0.986	-	1 h 9 m 30 s	1 h 21 m 36 s

The table summarizes the timings for convergence of PFClust with the different datasets. The total execution time typically includes four randomization and four clustering runs, but in the case of the 5000 2D vectors, the “0.99 average Rand Index between the four clusterings” criterion was not met until a fifth run had been carried out. The second column in the table shows the Rand Index of the final clustering against the original gold standard cluster definitions.

There exist cases when one wants to cluster large groups of data and time efficiency is very important. For those cases, a supervised mode of PFClust has been implemented in order to significantly speed up the process. The supervised mode of PFClust addresses the cost issue by applying an initial clustering on a training set and estimating a number of thresholds that would finally be applied to full dataset. The training set should be a small subset of the data, representing some coherent groups or clusters amongst the full dataset that we wish to cluster.

PFClust clusters the training set and uses the three best performing thresholds to estimate a total of nine threshold values (these are selected to allow for some variation). Then these nine values are applied on the full dataset and the clustering with the best Silhouette width is reported. Using the supervised version of the algorithm the time-consuming randomization step is removed, which results in a significant speed-up of the total process.

**Figure 8:**
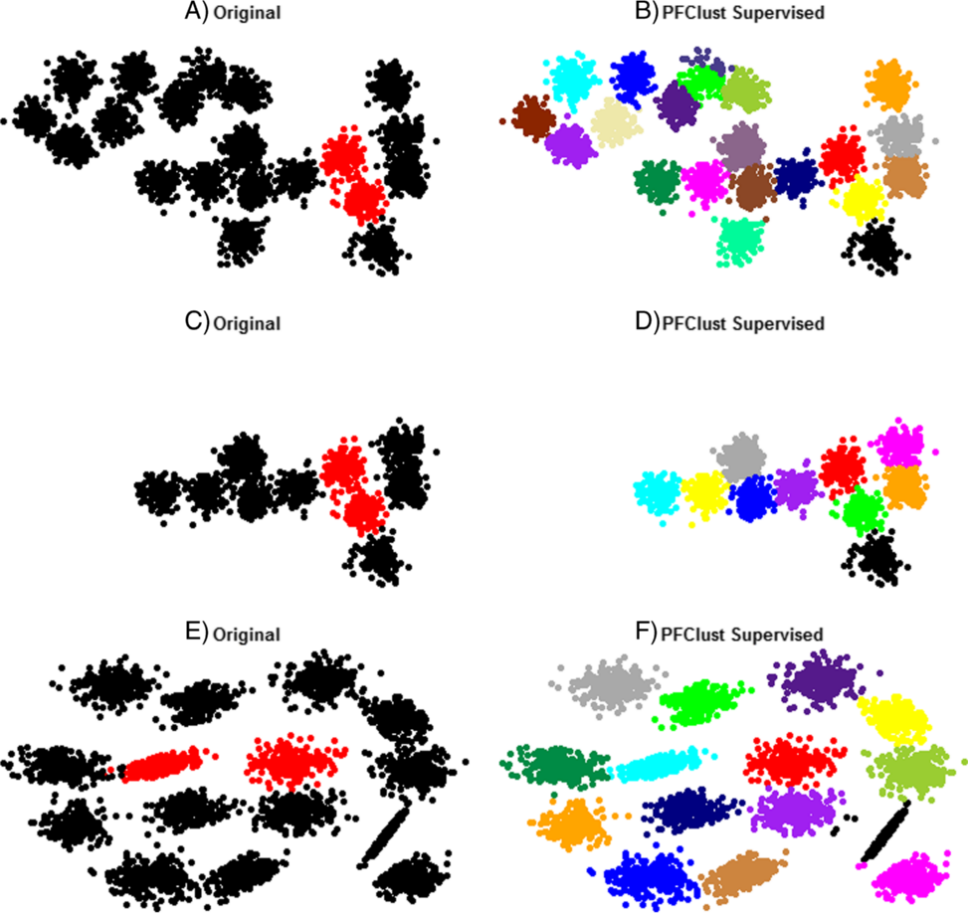
**Performance of PFClust supervised mode.** The 1500, 3000 and 5000 2D vector datasets clustered with PFClust supervised mode. Frames **A**, **C** &**E** show the original datasets, the training sets are coloured in red and the rest in black. Frames **B**, **D** &**F** show the clusterings generated by PFClust supervised mode.

As a validation of the supervised mode, we used the dataset with the 1500 vectors in 10 groups, the dataset of 3000 vectors distributed over 20 groups, and the larger one of 5000 vectors distributed over 15 groups. Figure 8 shows the original dataset and the split between training and full sets, as well as the resulting classification by PFClust. A training set of 300 points was selected and PFClust running using the supervised mode clustered the first two datasets very rapidly, compared to the unsupervised version, and with high accuracy. In more detail, for the dataset of 1500 vectors the algorithm took only three minutes to complete and gave an identical clustering to the unsupervised method. For the 3000 vector group it took only 20 minutes, again with a very good Rand Index of 0.951 compared with the original clustering, and in fact very slightly better than the 0.949 achieved by the unsupervised PFClust. Finally, for the larger group the algorithm used a training set of 626 vectors and took 1 hour and 21 minutes to complete, giving again a very good Rand Index of 0.986. In each case, the Rand Indices achieved by the supervised and unsupervised modes of PFClust are virtually identical, but the supervised mode is much faster.

### Protein fold clustering using polar Fourier expansions

**Figure 9:**
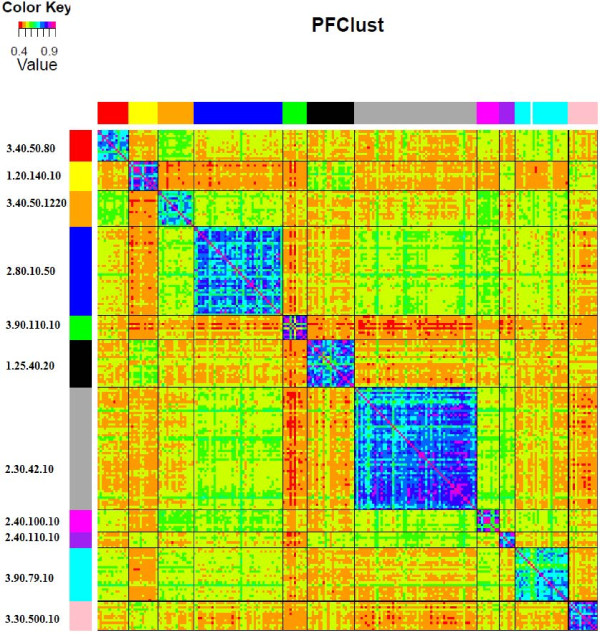
**Heat map of protein domain to protein domain density similarities.** On the row side, the protein domains are coloured according to the CATH classification; on the column side, the protein domains are coloured according to PFClust.

When we used PFClust to cluster the 224 protein domains in 11 CATH superfamilies, as shown in Table 4, the program reported 11 multi-member clusters and one singleton protein domain, i.e., 12 clusters in total. Figure 9 shows as a heat map the all-against-all similarity matrix of protein domains, each row is colour coded according to the CATH classification and each column according to the PFClust clustering.

**Table 4:** CATH superfamilies selected for the study

**Superfamily**	**Name**	**Members**	**Representative structure**
1.20.140.10	Butyryl-CoA Dehydrogenase, subunit A, domain 3	13	
1.25.40.20	-	21	
2.80.10.50	-	40	
2.30.42.10	-	55	
2.40.100.10	Cyclophilin	10	
2.40.110.10	Butyryl-CoA Dehydrogenase, subunit A, domain 2	7	
3.30.500.10	Murine Class I Major Histocompatibility Complex, H2-DB, subunit A, domain 1	13	
3.40.50.80	Nucleotide-binding domain of ferredoxin-NADP reductase (FNR) module	14	
3.40.50.1220	TPP-binding domain	16	
3.90.110.10	L-2-Hydroxyisocaproate Dehydrogenase, subunit A, domain 2	11	
3.90.79.10	Nucleoside Triphosphate Pyrophosphohydrolase	24	

The 11 CATH superfamilies that were selected to be clustered.

**Figure 10:**
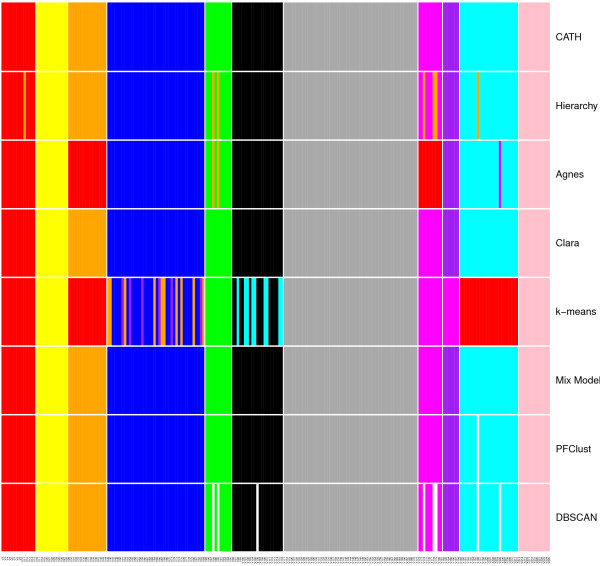
**Clustering comparison between the different algorithms and CATH.** The results of each of the different clustering algorithms as coloured lines, CATH colours represent different CATH superfamilies similarly to Figure 9.

The agreement between PFClust and the CATH classification is nearly perfect with a Rand Index of 0.996. There is only a minor difference between the original classification and the classification of PFClust, where the 1q27A00 protein domain was classified as a singleton, whereas CATH has it assigned to the 3.90.79.10 superfamily. We also tested the other clustering algorithms against this dataset and set the number of clusters to 11 for the five algorithms requiring this parameter. Table 2 summarizes the performance of each method, and Figure 10 visually shows the agreements and disagreements between the different clustering algorithms. We see that Mix Model and Clara are the top performing clustering algorithms, reproducing the exact CATH classification.

### Finding the correct number of clusters

**Figure 11:**
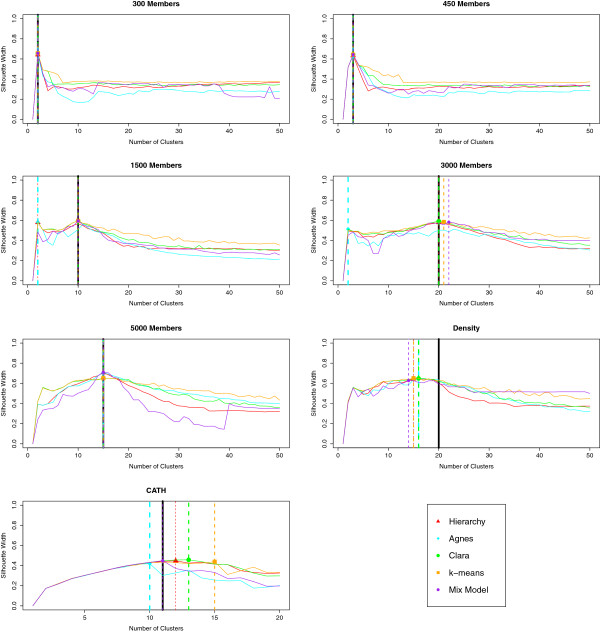
**Using Silhouette width to define the stopping rule.** As an addendum to the main work, we tested the use of the Silhouette width as a characteristic measure from which to decide the correct number of clusters. We ran the deterministic methods once each. We also ran the stochastic Clara and k-means algorithms 100 times each for every number of clusters, k, between 2 and 50. The run with the best Silhouette width for a given algorithm was selected, thus deciding the number of clusters to report.

For each of the aforementioned datasets, we tested the use of the Silhouette width as the criterion for identifying the number of clusters – similarly to the way we ran DBSCAN. Since all the algorithms depended on a single parameter *k* (number of clusters, inclusive of singletons), we varied this number from 2 to 50 and the results are shown in Figure 11. Note that these data are considered separately and do not contribute to the main results described previously, for which purpose the ‘correct’ number of clusters was instead passed to Hierarchy, Agnes, Clara, k-means and Mix Model as a parameter.

We see that in most cases, except for the density dataset, at least one method found the correct number of clusters by using the maximum Silhouette width as the stopping criterion. However, no method is consistently able to do this for the different datasets.

## Conclusions

It has been shown that PFClust can accurately group data according to their similarities without the need for any parameter tuning. Our clustering results on the synthetic datasets not only show that PFClust provides structurally meaningful clusters, but also that it performs best when compared to six other well-known clustering algorithms. Clustering protein domains using a density representation gives excellent agreement with the CATH part-manually curated classification. In the future, the full CATH database could be automatically clustered based on such density representations of protein domains.

## Competing interests

The authors have received funding from WADA. Other than this sponsorship, the authors declare no conflict of interest.

## Authors’ contributions

LM conceived and implemented the algorithm, carried out the experiments with PFClust and participated in the drafting of the manuscript. LM and NN designed the experimental and validation studies and carried out the comparison of PFClust with the other methods. NN participated in the drafting of the manuscript. JBOM participated in the drafting of the manuscript and provided guidance. All authors read and approved the final manuscript.

## Supplementary Material

Additional file 1: Figure S1 Comparison of different clustering algorithms; **Figure S2.** Comparison of the 300 and 450 2D vector datasets; **Figure S3.** Comparison of different clustering algorithms; **Figure S4.** Comparison of different clustering algorithms; **Figure S5.** Comparison of different clustering algorithms; **Table S1.** R packages used in the comparison of different clustering methodologies. Click here for file

## Availability

By request to the corresponding author, lazaros.mavridis.lm@gmail.com.
