# Supplementary material for: PFClust: a novel parameter free clustering algorithm
Source: BMC Bioinformatics. 2013 Jul 3;14:213. doi: 10.1186/1471-2105-14-213 (PMC3747858; doi:10.1186/1471-2105-14-213)
Supplement: Additional file 1: Figure S1 — Comparison of different clustering algorithms; Figure S2. Comparison of the 300 and 450 2D vector datasets; Figure S3. Comparison of different clustering algorithms; Figure S4. Comparison of different clustering algorithms; Figure S5. Comparison of different clustering algorithms; Table S1. R packages used in the comparison of different clustering methodologies. [file 1471-2105-14-213-S1.pdf]

PFClust: A Novel Parameter Free Clustering Algorithm

Lazaros Mavridis,[1*§] Neetika Nath[1*] and John B.O. Mitchell[1]

Biomedical Sciences Research Complex and EaStCHEM School of Chemistry, Purdie Building, University of St Andrews, North Haugh, St Andrews, Scotland, KY16 9ST, UK

Additional File 1

Supplementary Figures S1-S5 and Supplementary Table S1

Figure S1 – Comparison of different clustering algorithms

The 1500 vector dataset clustered with all seven different clustering algorithms. For each algorithm the points that are assigned to incorrect clusters are shown in red.

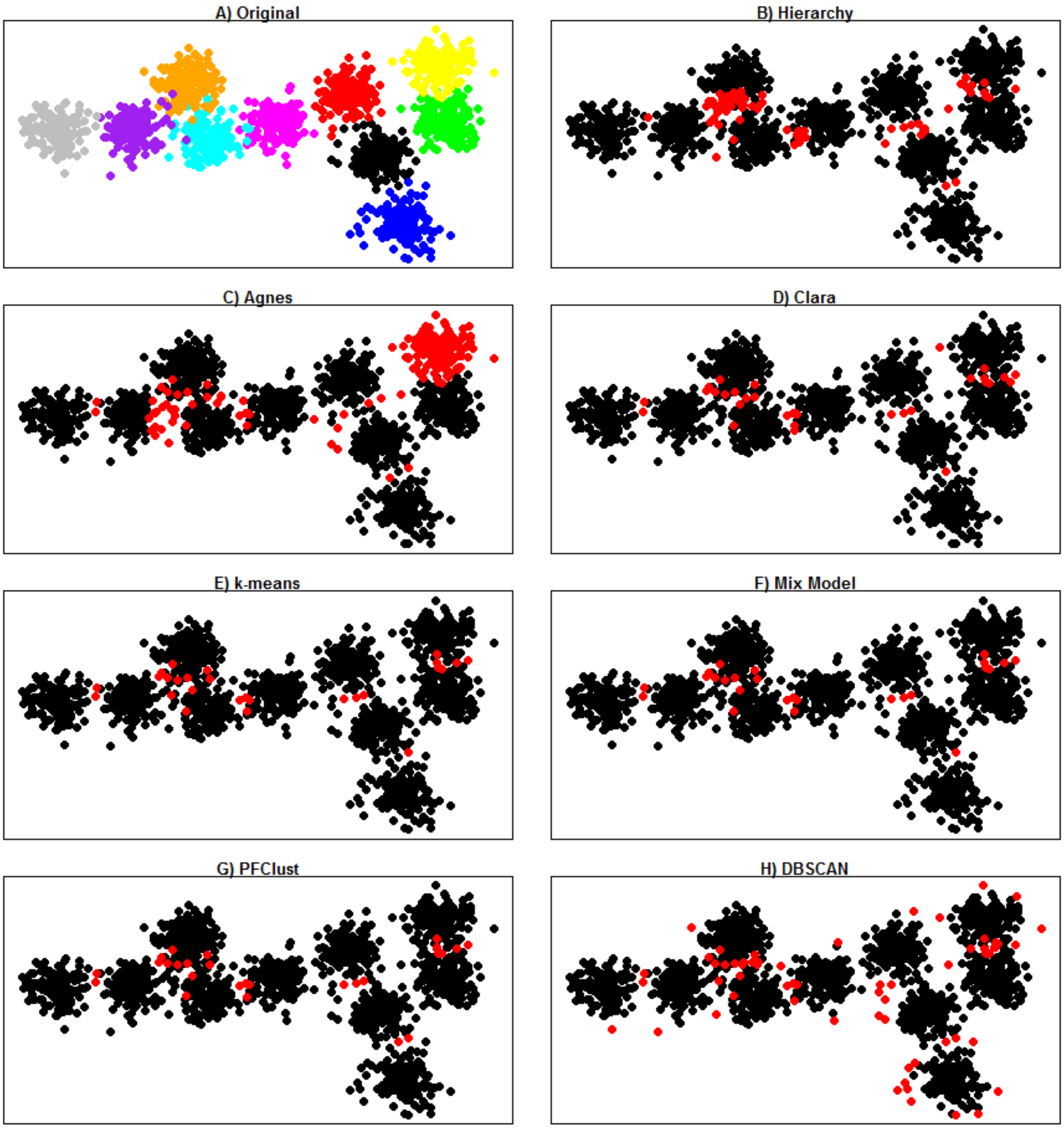

Figure S2 – Comparison of the 300 and 450 2D vector datasets

In frames A (300 2D vectors) and G (450 2D vectors), two of the datasets that were used and their original gold standard clusterings are shown. In frames B, C, D, E and F, the proposed clusterings by each method for the 300 2D vector dataset are given. In frames H, I, J, K and L, the proposed clusterings by each method are shown for the 450 2D vector dataset. For each algorithm the points that are assigned to incorrect clusters are shown in red.

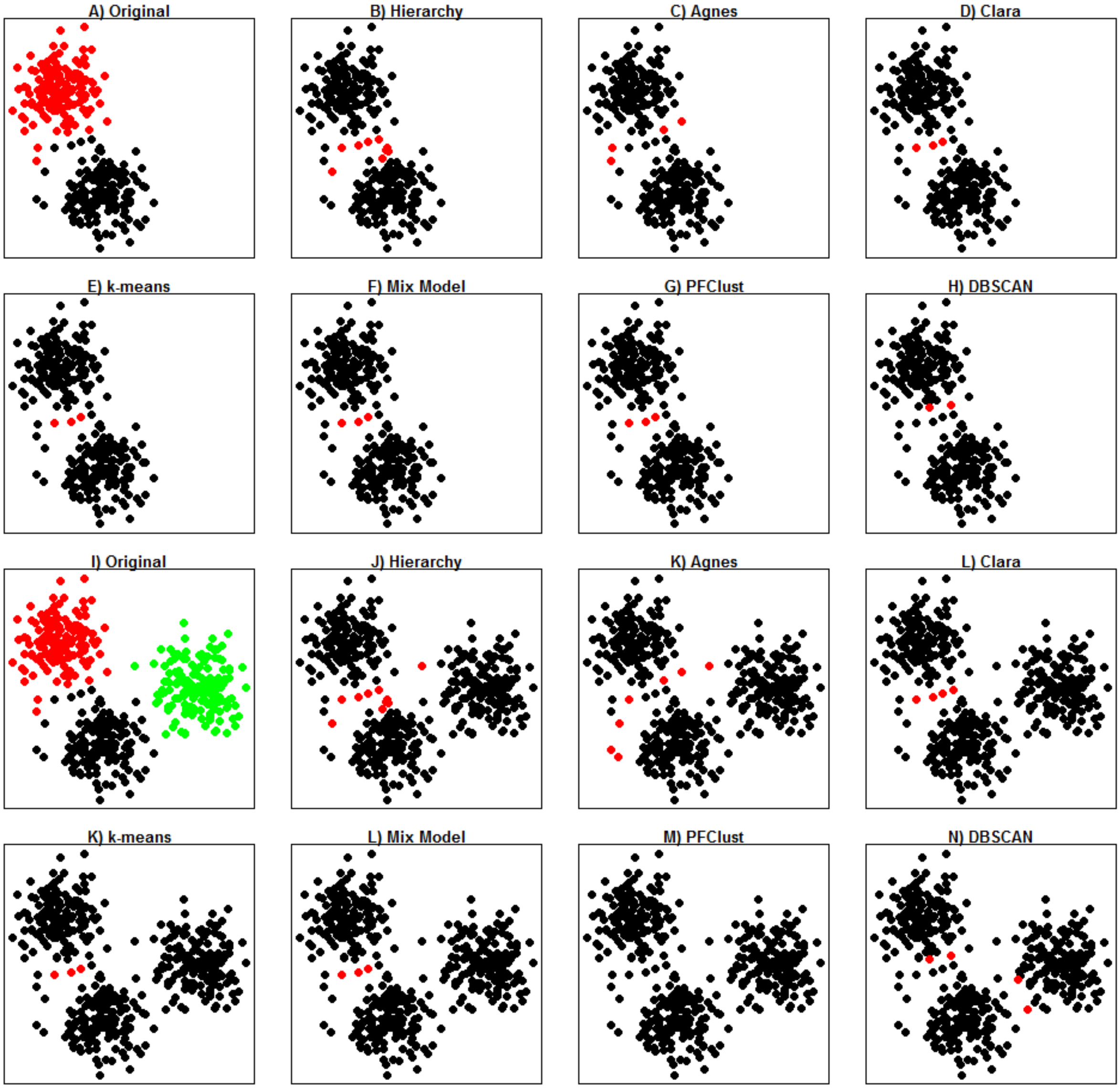

Figure S3 – Comparison of different clustering algorithms

The 3000 vector dataset clustered with all seven different clustering algorithms. For each algorithm, the points that are assigned to incorrect clusters are shown in red.

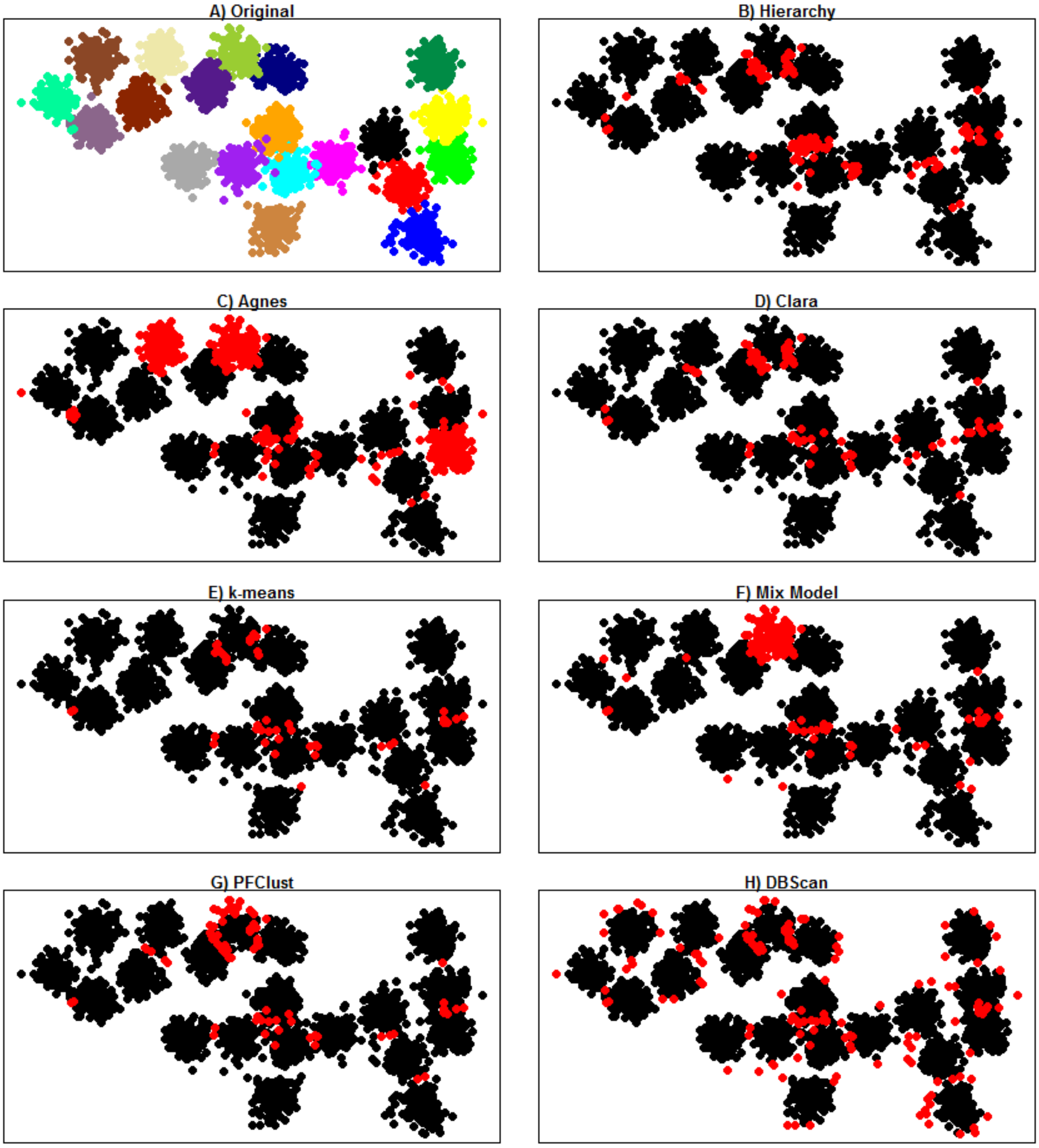

Figure S4 – Comparison of different clustering algorithms

The 5000 vector dataset clustered with all seven different clustering algorithms. For each algorithm the points that are assigned to incorrect clusters are shown in red.

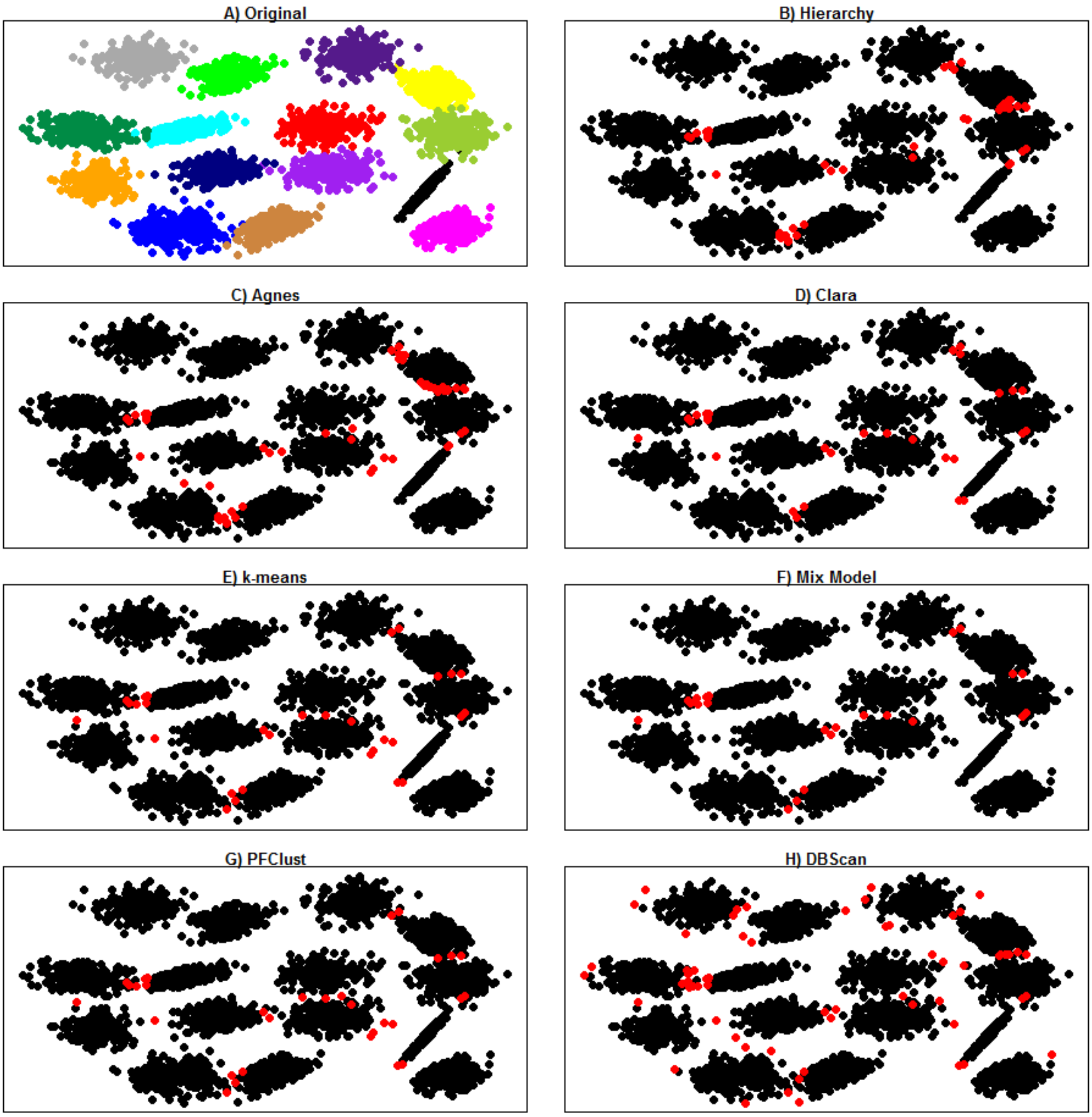

Figure S5 – Comparison of different clustering algorithms

The 928 vector dataset clustered with all seven different clustering algorithms. For each algorithm, the points that are assigned to incorrect clusters are shown in red.

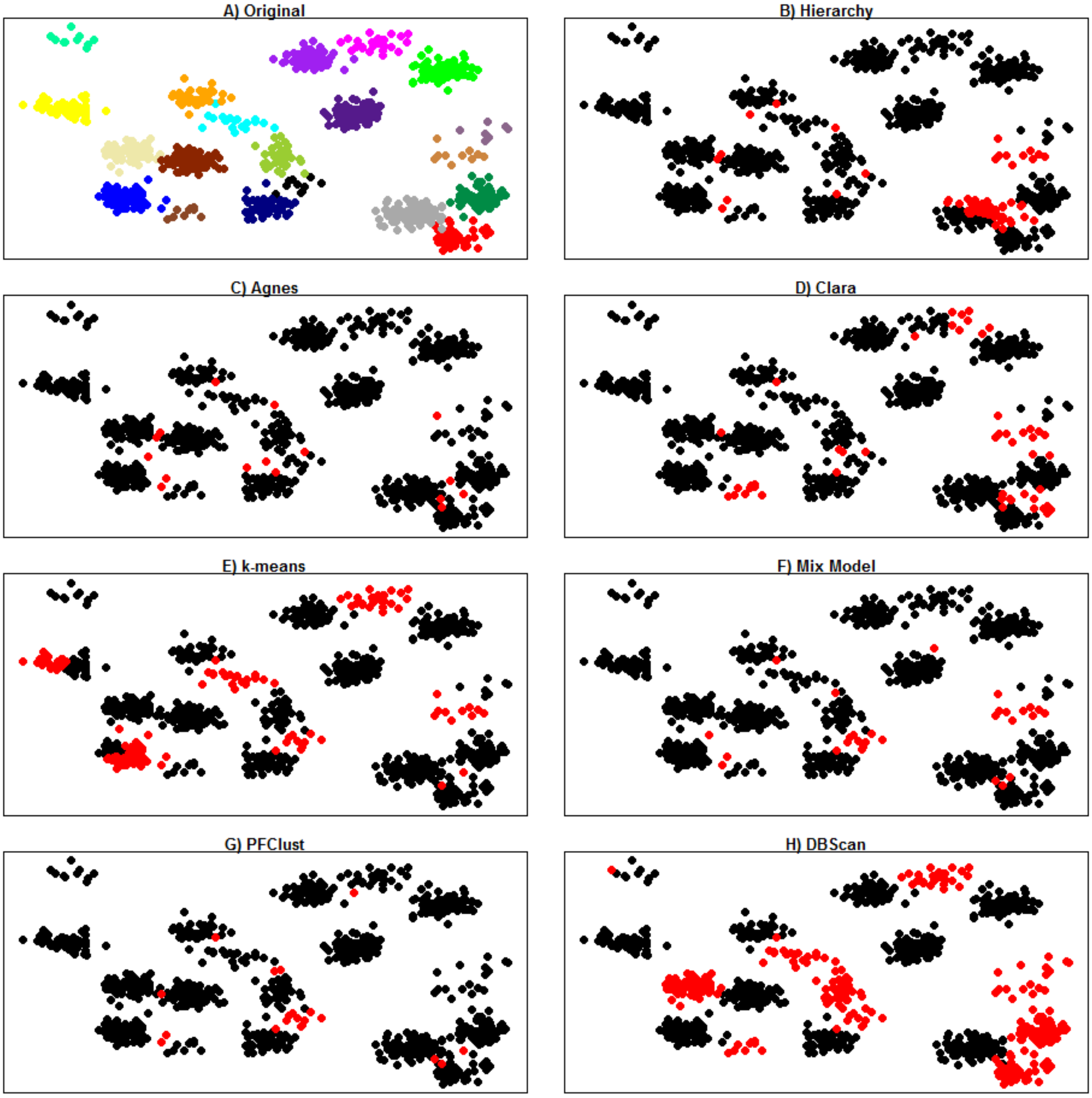

Table S1. R packages used in the comparison of different clustering methodologies.

Method	R Package
**Hierarchy**	**stats**
**Agnes**	**cluster**
**Clara**	**cluster**
**k-means**	**stats**
**Mix Model**	**Mclust**
**DBSCAN**	**FPC**
